# Antifungal Drugs: Special Problems Treating Central Nervous System Infections

**DOI:** 10.3390/jof5040097

**Published:** 2019-10-11

**Authors:** Elizabeth Dodds Ashley

**Affiliations:** Duke Center for Antimicrobial Stewardship and Infection Prevention, Duke University School of Medicine, Durham, NC 27710, USA; libby.dodds@duke.edu; Tel.: +1-919-681-3357

**Keywords:** antifungal, central nervous system, CSF

## Abstract

Treating fungal infections in the central nervous system (CNS) remains a challenge despite the availability of new antifungal agents. Therapy is limited by poor understanding of the kinetic properties of antifungal drugs in the CNS compounded by lack of data for many agents. In some cases, clinical response rates do not correspond to data on drug concentrations in the cerebral spinal fluid and/or brain parenchyma. In order to better characterize the use of antifungal agents in treating CNS infections, a review of the essential principles of neuroPK are reviewed. Specific data regarding antifungal drug concentrations in the cerebral spinal fluid and brain tissue are described from human data where available. Alternative dosing regimens and the role of antifungal drug concentration monitoring in treating fungal infections in the CNS are also discussed. Having a better understanding of these key concepts will help guide clinicians in determining the best treatment courses for patients with these devastating infections.

## 1. Introduction

Invasive fungal infections in the central nervous system (CNS) are particularly difficult to treat and are associated with high morbidity and mortality rates. These infections often attack patients immunosuppressed by underlying disease states, such as human immunodeficiency virus (HIV) or their treatments including antineoplastic agents. Recently, patients with less severe pathology receiving immune-modulating biologic agents have also emerged as a group at risk of CNS fungal infections.

Antifungal drug therapy is the cornerstone of management of fungal infections in the central nervous system. The unique challenges of drug penetration to the site and the variability of pharmacokinetic and dynamic characteristics between agents and individual patients further complicate the successful treatment of these life-threatening infections. This review summarizes many principles which should be considered when administering antifungal treatment for CNS disease.

## 2. NeuroPK

The key to determining the ability of an individual antifungal agent to treat infections of the CNS is describing drug exposure within the CNS or neuroPK. Similar to more traditional pharmacokinetics, this is a description of the absorption, distribution, metabolism and elimination of drug within the CNS. The ability to obtain repeated sampling, or any sampling at all from the CNS (including cerebral spinal fluid (CSF), interstitial fluid and brain tissue) of patients undergoing treatment significantly limits data to guide the best practices in this area.

The CSF is a protected site separated from traditional blood circulation by two primary barriers. The interstitial fluid of brain parenchyma is separated from blood circulation by the blood–brain barrier (BBB), while CSF is separated from blood circulation by the blood–CSF barrier (BCSFB) [1,2]. The latter is relatively more permeable than the BBB but provides little to no penetration into the brain parenchyma itself [3]. Additionally, the BBB provides nearly 5000 times the surface area of potential drug absorption and therefore is considered the main mechanism for brain drug exposure [4]. However, simply knowing the penetration of a drug into the CSF does not adequately predict the ability of any drug to reach the targeted infection site in the CSF. 

There are several drug characteristics that are associated with optimal entry into the CNS. These include lipophilicity, which allows maximal penetration through the lipid bilayer surrounding the CNS. Lipophilicity is measured as the octanol-water partition coefficient and is expressed as log P. Values between 0 to 1 are considered optimal for CNS penetration [5]. The second characteristic relates to protein binding as generally, only a free drug is available to cross into the CNS. It should be noted that lipophilicity itself often creates a high likelihood of protein binding and therefore, these factors may work in opposition in vitro, further complicating the ability to predict the ability of a particular drug to penetrate the CNS. Smaller molecules are more successful in crossing the tight junctions of the BBB and although sources differ, it appears that molecules with a molecular weight of less than 450 are optimal [2]. Another indicator that a drug has a broad distribution that may include the CNS is a volume of distribution (Vd) close to 1 L/kg and some advocate for this as a marker for candidate drugs with CNS penetration during drug development [2]. Lastly, drugs that have a high affinity for efflux pumps, markedly p-glycoprotein can be easily removed from the CNS. Optimal therapies targeting the CNS would have a low affinity for these transports, resulting in improved CNS exposure [2,4]. A summary of antifungal drugs displaying each of these characteristics appears in Table 1. 

Distribution within the CNS is a key component to ultimate drug availability at the target site. There is no restrictive barrier between interstitial fluid of the brain parenchyma and the CSF, therefore, once penetrating the CNS, the drug can move fluidly between these spaces [14]. However, drug concentrations often do not equilibrate between these spaces due to many factors, including drug clearance. Debate remains as to whether CSF concentrations alone are sufficient predictors of drug availability within the CNS [2,3]. Variable drug concentrations have been noted between different areas of the CSF, particularly lumbar and ventricular CSF, whereby in the setting of therapeutic drug concentrations in the lumbar CSF, no drug could be detected in ventricular fluids [15,16].

Drug clearance from the CNS can also affect drug availability. A drug is cleared from the CNS by two main mechanisms: reverse diffusion across the blood brain barrier, often employing transport mechanisms, and drainage of interstitial fluid into venous blood [2]. The latter accounts for the majority of drug clearance from the CNS and typically occurs at a rate greater than drug entry into the CNS. Of the transport mechanisms in the CNS, p-glycoprotein is perhaps the most relevant efflux method for antifungal drug exposure although carrier-assisted transport does also occur [1]. Although small amounts of drug can be metabolized within the BBB, this is not a significant contributor to drug removal from the CNS.

As with traditional pharmacokinetic principles, many factors can alter neuroPK parameters. These include patient factors associated with underlying disease, such as immunosuppression, altered protein binding secondary to protein deficiency, and changes in drainage rates into venous blood by concomitant medications [2]. CNS permeability also varies with age with the very young and elderly demonstrating enhanced permeability [1]. Many infection-related parameters impact kinetic properties and these may change during the course of infection treatment [2].

One of the most discussed infection-related parameters affecting CNS drug exposure is meningeal inflammation. It is thought that inflammation results in a leakier BBB and decreases return clearance into venous blood [17,18]. Additionally, efflux pumps, such as p-glycoprotein can see reduced activity during times of inflammation [19]. Altogether, these factors can help to enhance drug concentrations in the CNS during infection but can change during treatment as patients respond and symptoms including inflammation are resolved.

## 3. Antifungal Therapy in the Central Nervous System

Understanding the neurologic pharmacokinetic effects of the various antifungal agents allows for better prediction of which agents will allow adequate CNS exposure to treat fungal disease at this sequestered site. However, treatment recommendations and guidelines for managing CNS fungal infection, as discussed throughout the rest of this issue, place much greater weight on clinical experience for managing the disease. 

Data regarding CNS penetration from human (where available) and animal models are depicted in Table 2. As previously mentioned, even agents with nearly undetectable drug concentrations either in brain tissue or CSF have been associated with successful clinical outcomes. 

Amphotericin B and associated lipid formulations remain the cornerstone of treatment for many CNS fungal infections [20]. Data has emerged that perhaps liposomal amphotericin B (L-Amb) is the preferred lipid formulation when treating CNS disease, but this fact is a matter of debate [21]. Amphotericin B is often combined with flucytosine in this setting. Flucytosine has excellent CNS exposure but should not be used as monotherapy due to the rapid development of resistance [22]. 

Similarly, there is significant clinical experience with fluconazole for treating CNS disease based on the favorable kinetic profile in the CNS for this agent. Recently, voriconazole has also emerged as a reasonable treatment option for CNS disease and has been associated with successful treatment in many patient populations [23,24].

Many other newer antifungal agents lack experience in treating CNS disease and do not offer adequate CNS exposure to justify veering away from current standards of care. This is especially true for the echinocandin agents that have isolated evidence of successful treatment, but only at doses higher than those traditionally given and/or in combination with other agents [1,2]. Nearly all cases of CNS infection treatment with echinocandin occur in patients who have failed more standard therapies. Recent evidence suggesting enhanced CNS penetration seen in neonates may permit successful treatment of CNS infection with echinocandins, but at present, there is no adequate data to endorse this therapy [25]. Similarly, the newer azoles, isavuconazole and posaconazole, do not adequately penetrate the CNS and are not routinely used to treat fungal infections in the CNS.

The other articles in this issue focus on treating specific fungal infections in the CNS and provide greater detail on agent selection and dosing.

## 4. Direct Administration to the CNS

Given the limitations of a systemic drug reaching the CNS, direct administration of antifungal agents into the CNS has been attempted. The majority of data with this approach is with administration of amphotericin B deoxycholate via the intrathecal route [56,57]. Recent experience has been gained with the lipid-based amphotericin B formulations [58,59] and administration of the deoxycholate preparation using implanted drug reservoirs [60]. 

Intra-ventricular administration may help mitigate differential CSF drug distribution between lumbar and ventricular CSF, as previously described. This can be accomplished by administration via an Ommaya™ reservoir, a device invented to circumvent the limitation of lumbar drug administration for the treatment of CNS disease [61]. Using this approach also allows drug CSF to be removed before injecting a drug, which limits the concern for increased pressures from added drug volume. Although not standard practice, direct CNS administration of other antifungal drugs has been performed including intraventricular caspofungin [62].

Direct administration to the site of infection certainly has many theoretical advantages, including eliminating the complex issues surrounding CNS penetration. This circumvents the need for drug absorption into the CNS but does not change the other pharmacokinetic considerations required of antifungal therapy in the CNS. Most notably, drug elimination through routine CSF draining to the venous blood supply can lead to quickly declining drug concentrations [63]. Toxicities of direct drug administration in the CSF must also be considered. In the case of amphotericin B, nausea, vomiting and headache are most commonly reported, although more severe cases of neurotoxicity, including hearing loss and ataxia, have also been reported [57]. Therefore, to ensure optimal exposure this route of administration should be accompanied by systemic antifungal therapy. 

## 5. Therapeutic Drug Monitoring

Monitoring drug concentrations for antifungal drugs has become more common practice over the past decade [64]. For all patients being treated for central nervous system infections, serum drug concentration monitoring is recommended for targeted agents. In most cases it is not clinically practical to measure local drug concentrations within the CSF. In fact, most of the available human data on drug exposure in CNS fungal infections is a convenience sample of patients undergoing surgery as part of routine care or from samples obtained post-mortem [8]. Routine monitoring may be possible in patients with indwelling CSF shunts and may be helpful to determine CNS drug exposure but are not routinely recommended at this time.

## 6. Conclusions

There are several antifungal agents that can successfully be used to treat CNS fungal infections today. However, each antifungal agent possesses unique kinetic and dynamic properties that impact its ability to successfully treat infections in the CNS. Understanding key considerations to the challenge of delivering antifungal drugs to the CNS is essential for the successful treatment of this patient population.

## Figures and Tables

**Table 1:** Select properties associated with maximal CNS exposure [2,6].

Property	Antifungal Drugs Demonstrating Favorable Characteristics	References
High Lipophilicity	AmB-d ^1^, ABLC ^2^, L-AmB ^3^, anidulafungin	[7]
Low Protein Binding	Fluconazole, Voriconazole, Flucytosine	[8,9]
Low Molecular Weight (<450)	Isavuconazole, fluconazole, voriconazole, flucytosine	[7,8,10]
Not P-glycoprotein (efflux pump) substrate	isavuconazole flucytosine, AmB-d1, ABLC ^2^, L-AmB ^3^, caspofungin, micafungin, anidulafungin	[11,12]
Volume of Distribution around 1 L/kg	Fluconazole, AmB-d ^1^, ABLC ^2^, flucytosine, anidulafungin	[9,13]

^1^ AmB-d = amphotericin B deoxycholate, ^2^ ABLC = amphotericin B lipid complex, ^3^ L-AmB = liposomal amphotericin B.

**Table 2:** Antifungal drug penetration in the central nervous system (as % of serum concentration).

Drug	CSF Penetration	Brain Tissue Penetration	Clinical Success Reported	Reference(s)
Azole Agents				
Fluconazole	52–100%	70-≥100%	Yes	[26,27,28]
Itraconazole	<1%	50-≥100%	Yes	[8,29,30]
Isavuconazole	<1%	100–200%	Yes	[31,32,33,34,35]
Posaconazole	0–250%	50–90%	Yes	[36,37,38,39,40]
Voriconazole	22–100%	50->100%	Yes	[8,23,41,42,43]
Echinocandin Agents				
Anidulafungin	<1%	<1%	No	[44,45,46]
Caspofungin	<1%	<1%	High dose	[8,47,48]
Micafungin	<1%	<1–25%	No	[49,50,51]
Other Agents				
Amphotericin B (AmB-d) ^1^	<1%	27%	Yes	[21,52]
Amphotericin B lipid complex (ABLC) ^2^	<1%	22%	Yes	[21,53]
Liposomal amphotericin B (L-AmB) ^3^	<1%	3%	Yes	[21,54]
Flucytosine	75%	N/A	Yes	[52,55]

^1^ AmB-d = amphotericin B deoxycholate, ^2^ ABLC = amphotericin B lipid complex, ^3^ L-AmB = liposomal amphotericin B.
